# Supplementary material for: Integrated assessment modeling of a zero-emissions global transportation sector
Source: Nat Commun. 2024 May 24;15:4439. doi: 10.1038/s41467-024-48424-9 (PMC11126718; doi:10.1038/s41467-024-48424-9)
Supplement: Supplementary file 1 — Supplementary Information [file 41467_2024_48424_MOESM1_ESM.pdf]

Supplementary Information for

Integrated assessment modeling of a zero-emissions global transportation sector

Simone Speizer, Jay Fuhrman, Laura Aldrete, Mel George, Page Kyle, Seth Monteith, Haewon McJeon

Contents

Supplementary Figures 2
Supplementary Tables 15
Supplementary Methods 20
Supplementary Notes 21
Supplementary References 23

Supplementary Figures

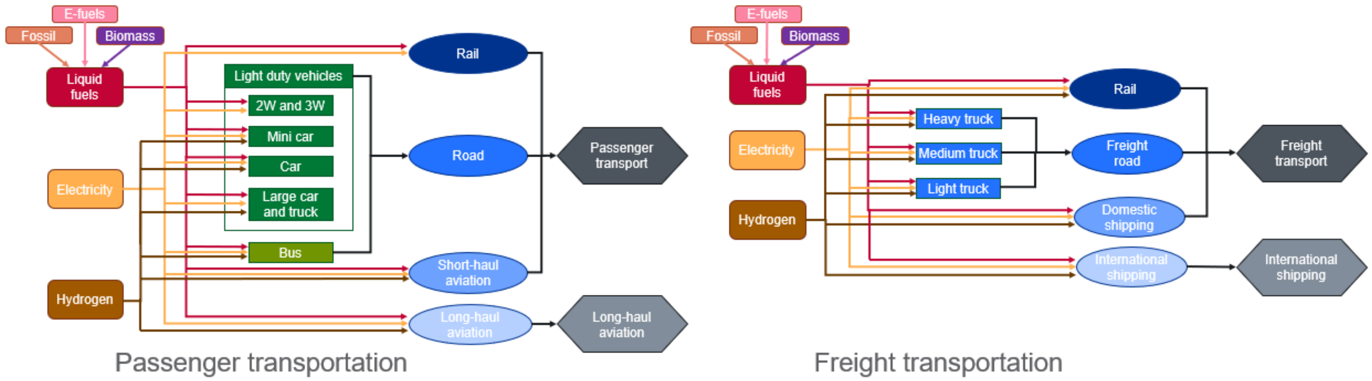

Supplementary Figure 1. GCAM transportation sector structure. The structure of the transportation sector in GCAM, including key fuel options for the sector. The nesting structure allows for mode-shifting—alongside fuel shifting—between grouped modes or sub-modes in response to climate policies. International shipping and long-haul aviation are grouped separately from all other freight and passenger modes. We show the primary fuel options for transport, across all modes, in this figure; in some regions, coal is also a fuel choice for freight rail, as is natural gas for passenger road modes and light freight trucks. 2W = two wheel, 3W = three wheel.

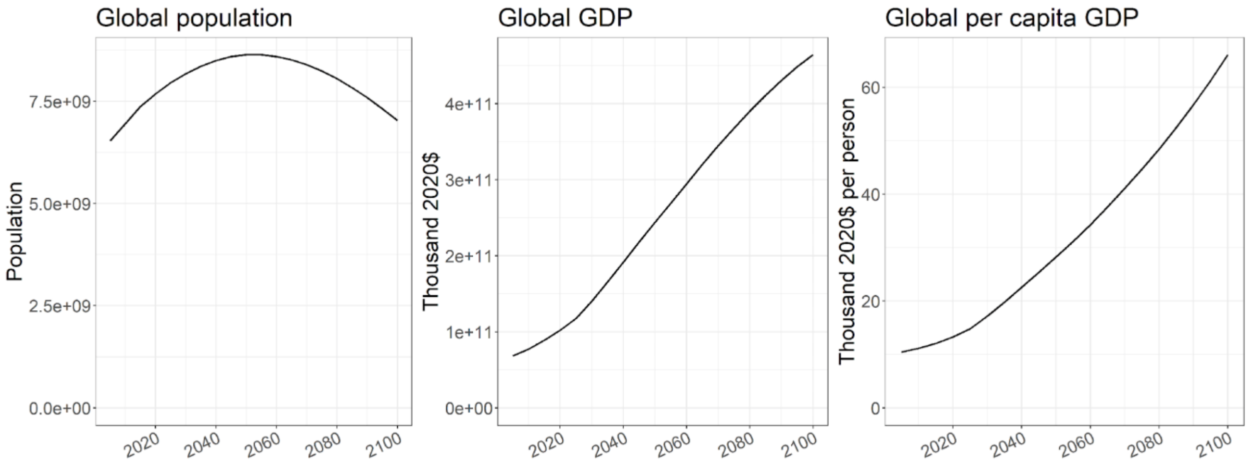

Supplementary Figure 2. Population and GDP. Population and gross domestic product (GDP) assumptions used in all primary scenarios, aligned with Shared Socioeconomic Pathway 1.

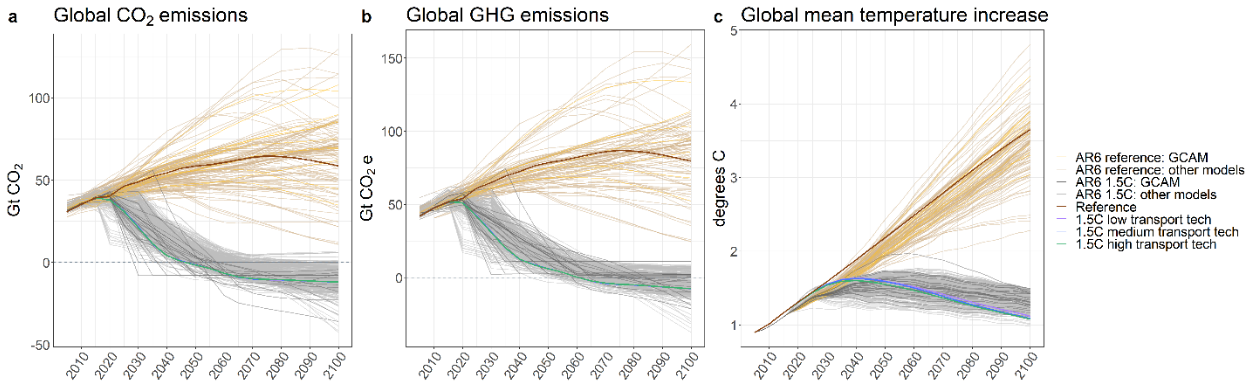

Supplementary Figure 3. Total CO_2 and GHG emissions and temperature change. (a) Global CO_2 emissions, including both fossil fuel and industry sources and land use change, (b) global total greenhouse gas (GHG) emissions, and (c) the resulting global mean temperature increase, in the four primary scenarios in our analysis compared to the scenarios in the IPCC Sixth Assessment Report (AR6) database that are consistent with limiting end-of-century warming to 1.5°C (gray) and that represent a continuation of baseline trajectories (brown). Scenarios from the AR6 database that were generated by GCAM are shown in brighter colors, dark gray for the 1.5°C-consistent scenarios and golden brown for the baseline scenarios. Note that the GCAM scenarios in the AR6 database employed earlier versions of GCAM than the version used in this study, and thus the scenarios may differ in historical periods due to differences in calibration data and other model updates. Global warming potentials used for the non-CO_2 greenhouse gases are from the AR5 report. The 50th percentile FaIRv1.6.2 surface temperature variable is used for global mean temperature increase for the AR6 scenarios. For the scenarios from this study, global mean temperature change reported in the figure comes from Hector, the reduced form climate model linked to GCAM.

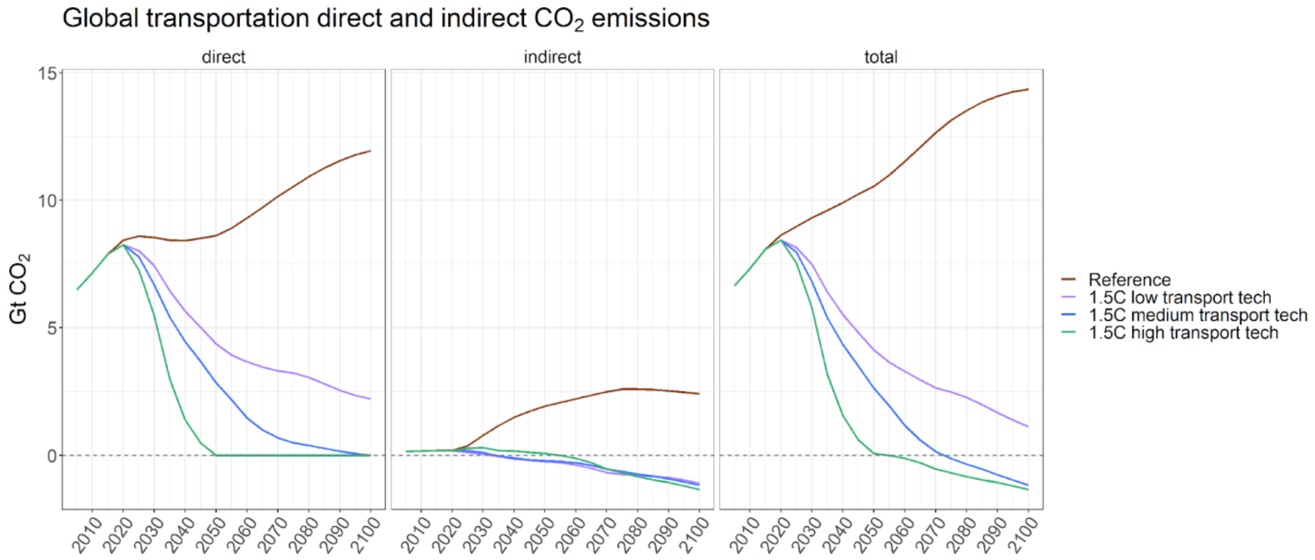

Supplementary Figure 4. Transportation direct and indirect emissions. Global direct and indirect CO_2 emissions from the transportation sector in the primary scenarios.

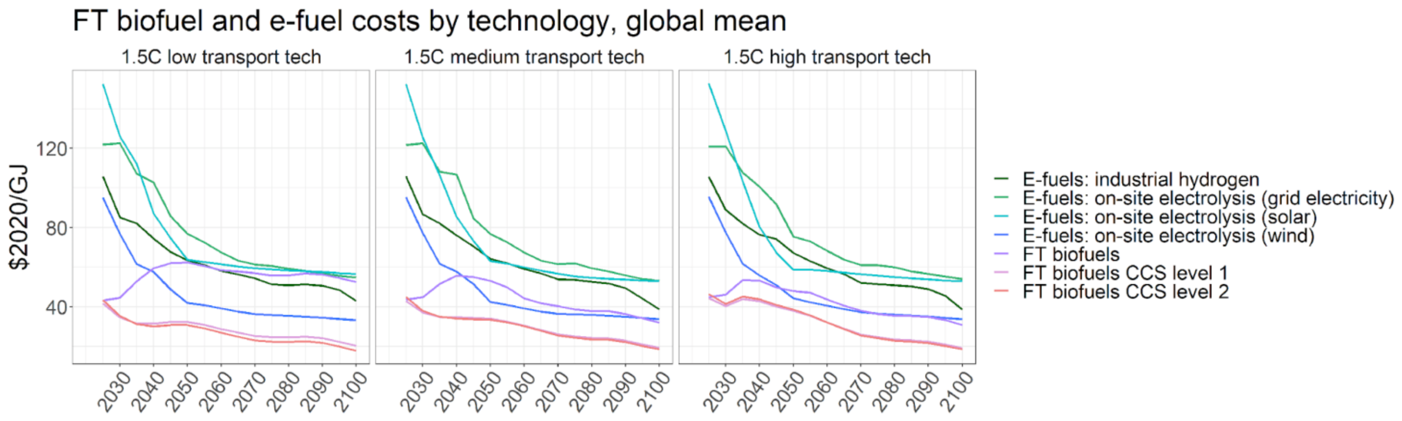

Supplementary Figure 5. FT biofuel and e-fuel costs. Global mean of Fischer-Tropsch (FT) biofuel and e-fuel costs by production technology. The different carbon capture and storage (CCS) levels indicate varied carbon removal fractions, with level 2 technologies employing carbon capture on a more expansive set of CO_2 sources and thus yielding a higher removal fraction than level 1 technologies. The costs shown in this figure incorporate the effects of carbon prices on the overall cost of the fuel produced.

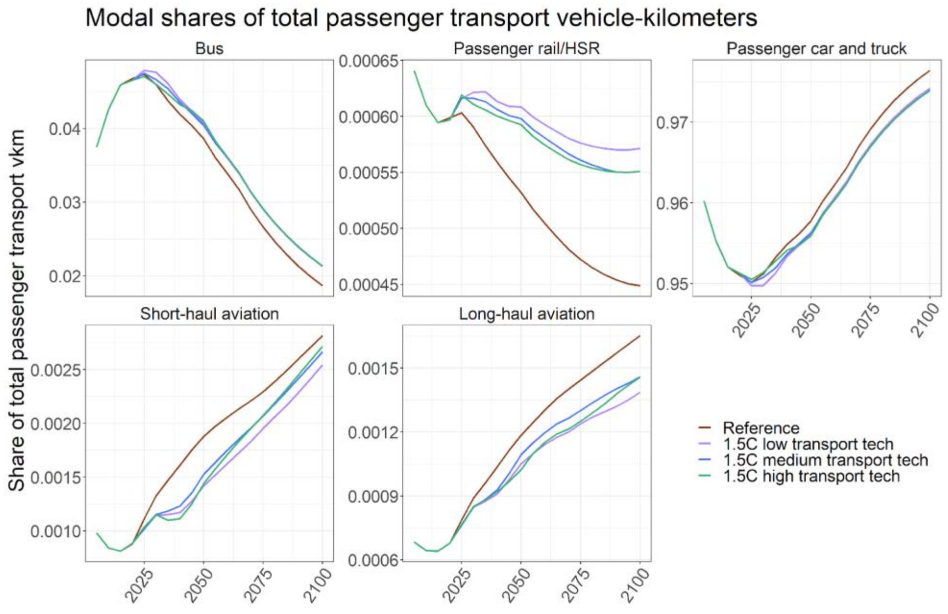

Supplementary Figure 6. Modal shares of total passenger transport vehicle-kilometers. Shares of total passenger transport vehicle-kilometers (vkm) provided by each mode in the four scenarios. In the decarbonization scenarios, the elevated preference for public transit increases the shares of vehicle-kilometers provided by buses and rail and decreases the shares provided by other modes relative to the reference scenario. Higher ambition in the transport sector increases the shares of vehicle-kilometers provided by difficult to decarbonize modes, particularly aviation. HSR = high-speed rail.

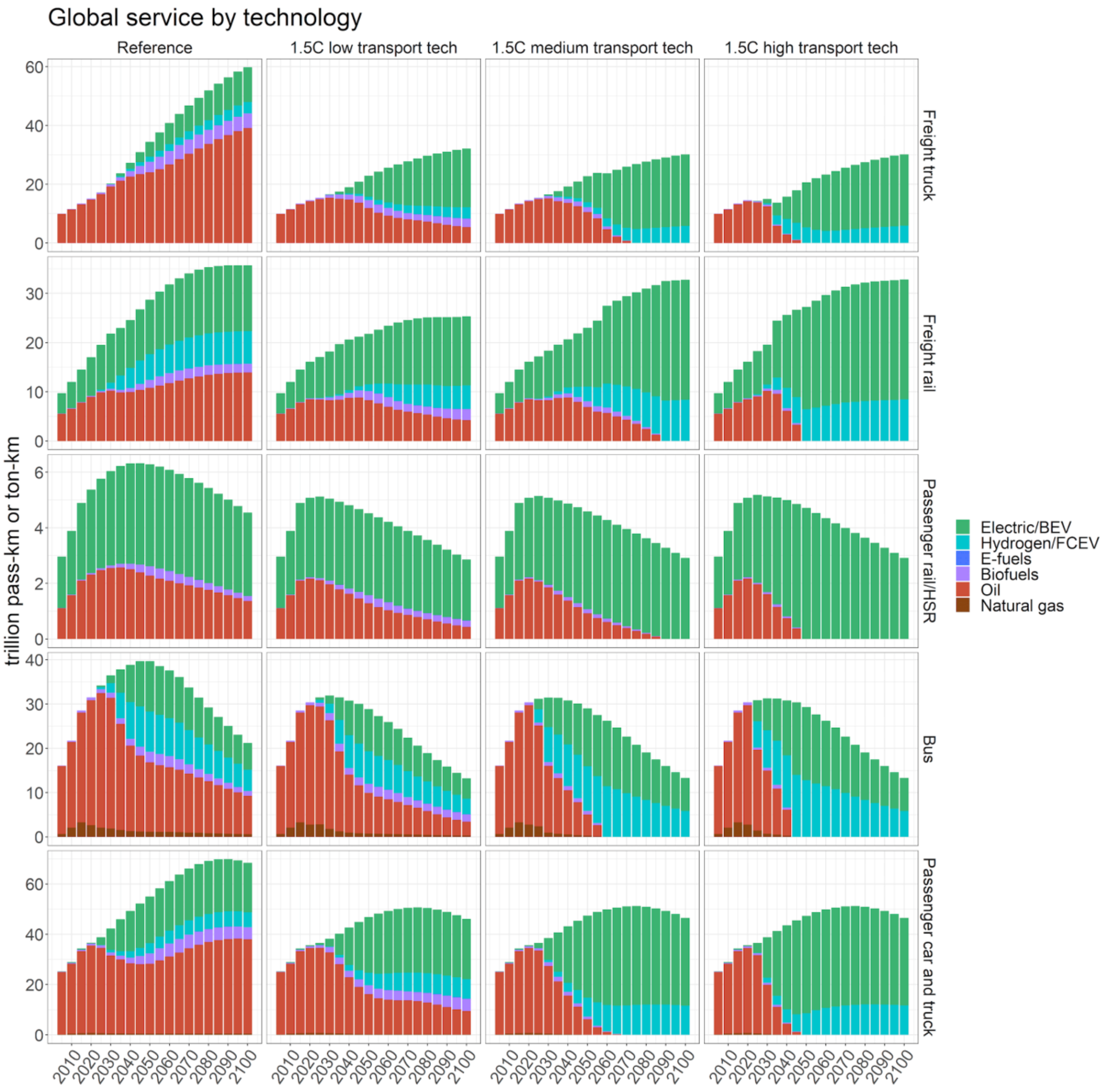

Supplementary Figure 7. Transportation service by technology for modes other than aviation and shipping. Global service provided by technology for freight trucks, freight rail, passenger rail and high-speed rail (HSR), buses, and passenger cars and trucks across the four scenarios. Note the different y-axis scales and units for each mode; units are trillion passenger-kilometers (pass-km) for passenger modes and trillion ton-kilometers (ton-km) for freight modes. Also note that there is a very small amount of coal-based freight rail service that is not shown in the figure as it phases out by 2025 and provides only a miniscule contribution to global freight rail transport in the preceding periods (0.009 trillion ton-km of service in 2005, 0.007 trillion ton-km in 2010, and 0.002 trillion ton-km in 2015 and 2020). BEV = battery electric vehicle, FCEV = fuel cell electric vehicle.

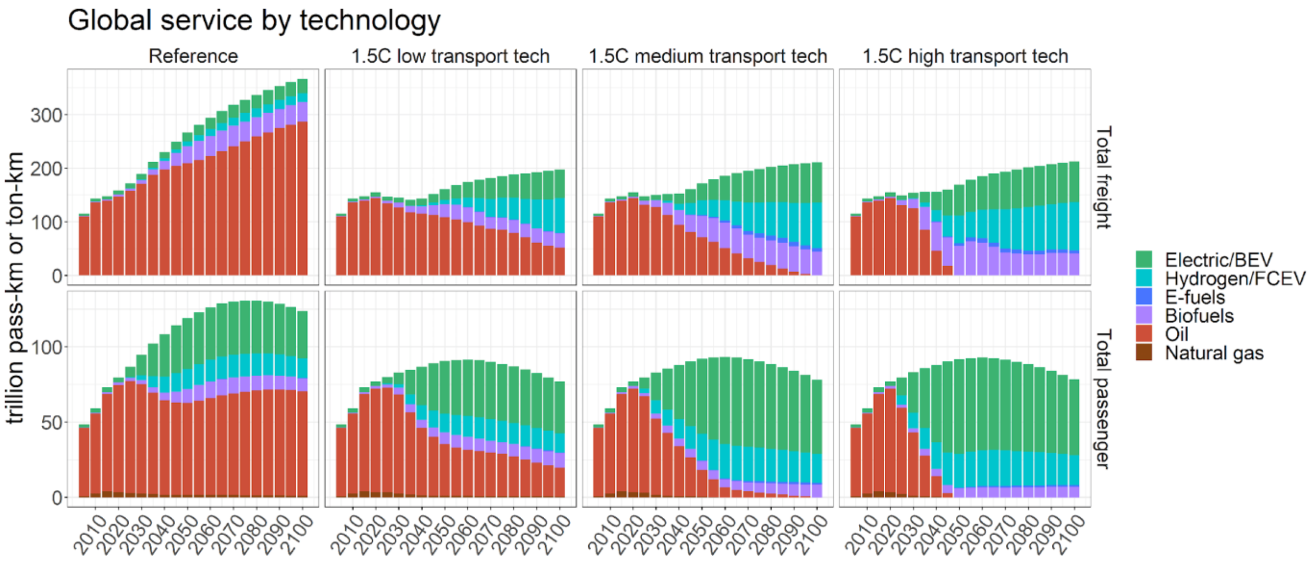

Supplementary Figure 8. Total transportation service by technology. Global service provided by technology for the full transportation sector, separated into freight transport and passenger transport, across the four scenarios. Note the different y-axis scales and units; units are trillion passenger-kilometers (pass-km) for passenger transport and trillion ton-kilometers (ton-km) for freight transport. Also note that there is a very small amount of coal-based freight service (exclusively freight rail) that is not shown in the figure as it phases out by 2025 and provides only a miniscule contribution to global freight transport in the preceding periods (0.009 trillion ton-km of service in 2005, 0.007 trillion ton-km in 2010, and 0.002 trillion ton-km in 2015 and 2020). BEV = battery electric vehicle, FCEV = fuel cell electric vehicle.

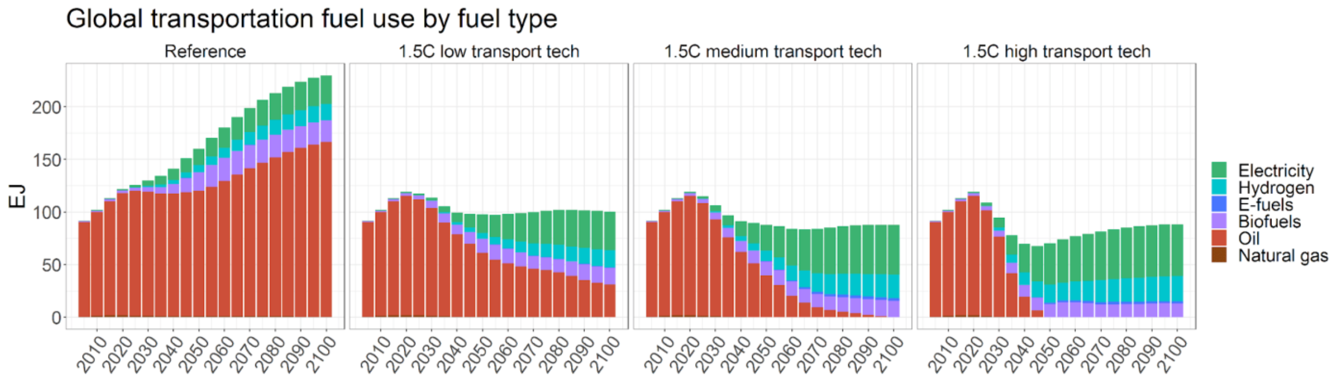

Supplementary Figure 9. Transportation total fuel use. Global fuel use by the transportation sector across the four scenarios. Note that there is a very small amount of coal use in transport, exclusively utilized by freight rail prior to 2025, which is not shown in the figure as it provides only a miniscule contribution to global transport fuel use (0.011 EJ in 2005, 0.007 EJ in 2010, and 0.002 EJ in 2015 and 2020).

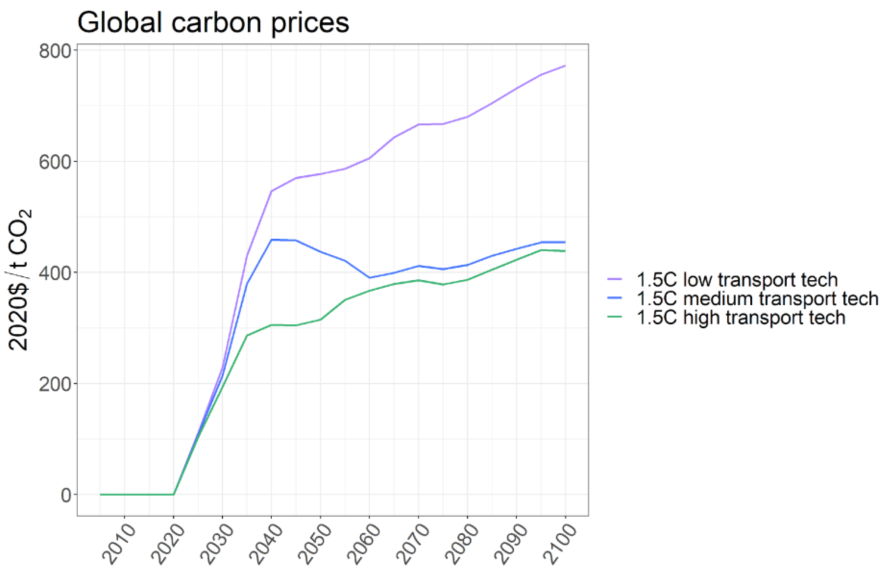

Supplementary Figure 10. Carbon prices across decarbonization scenarios. Global carbon prices, calculated endogenously by GCAM as required to meet the carbon emissions constraint, across decarbonization scenarios. Units are 2020 USD per ton of CO_2.

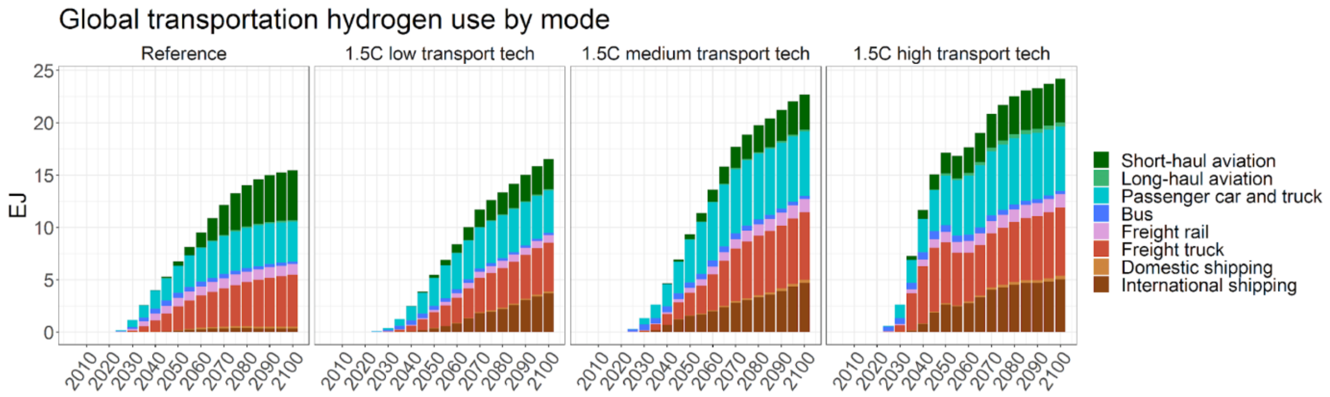

Supplementary Figure 11. Transportation hydrogen use by mode. Global hydrogen consumption by transportation modes across the four scenarios.

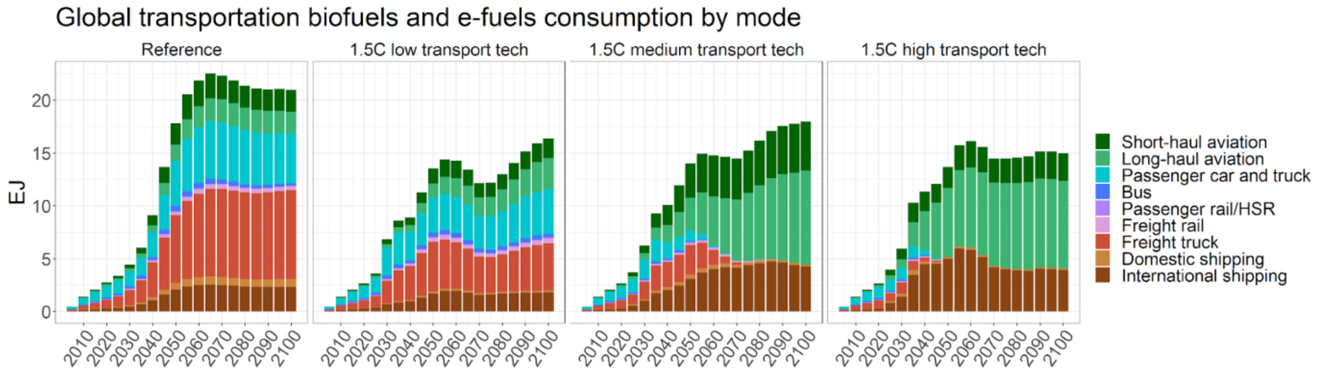

Supplementary Figure 12. Transportation biofuels and e-fuels use by mode. Global aggregated biofuels and e-fuels consumption by transportation modes across the four scenarios. HSR = high-speed rail.

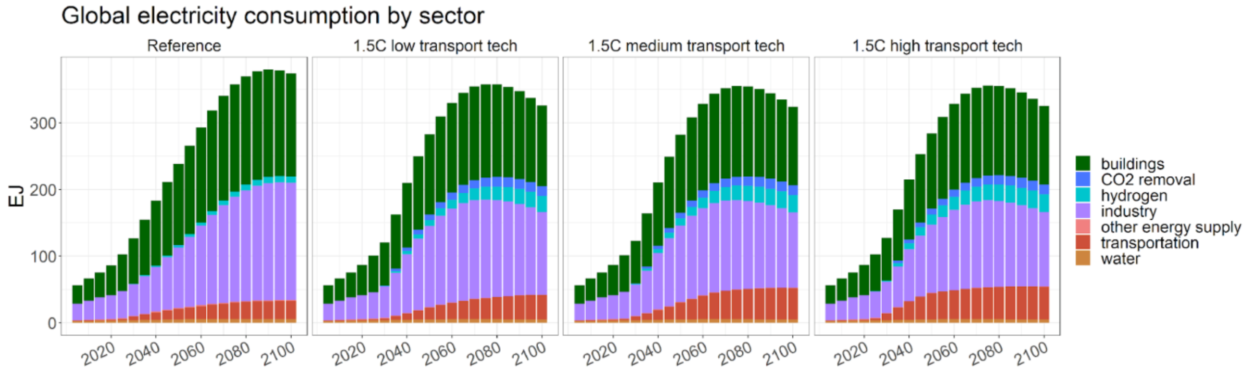

Supplementary Figure 13. Global final electricity consumption by sector. Global electricity consumption by sector across the four scenarios. Note that only final electricity consumption is shown in the figure.

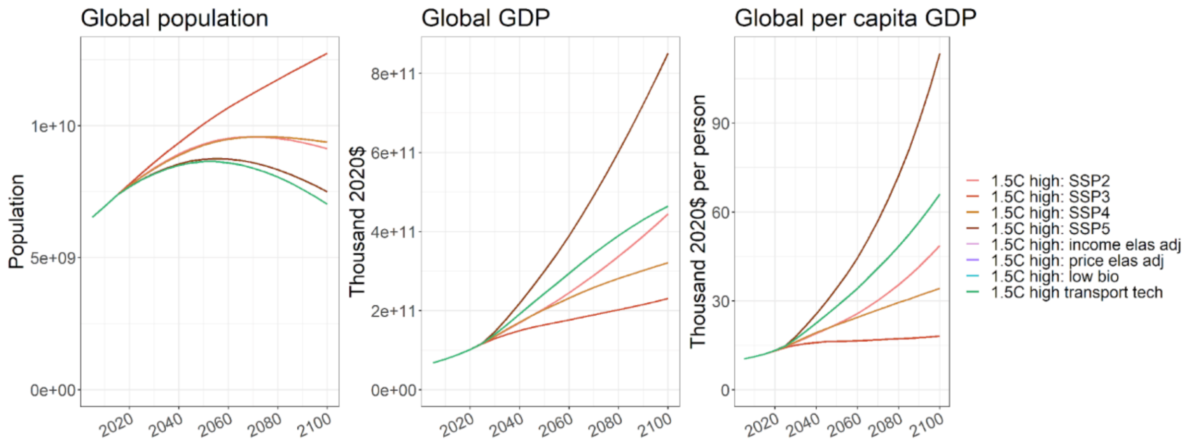

Supplementary Figure 14. Population and GDP, sensitivity scenarios. Population and gross domestic product (GDP) assumptions used in the sensitivity scenarios and the standard high transportation technology scenario, aligned with the Shared Socioeconomic Pathways (SSP) trajectories. Note that the income elasticity adjustment scenario, the price elasticity adjustment scenario, and the low bio scenario employ the same population and GDP assumptions as the standard high transportation technology scenario (all aligned with SSP1), and thus these scenarios do not appear visibly in the figure. Income elas adj = income elasticity adjustment, price elas adj = price elasticity adjustment, low bio = low bioenergy.

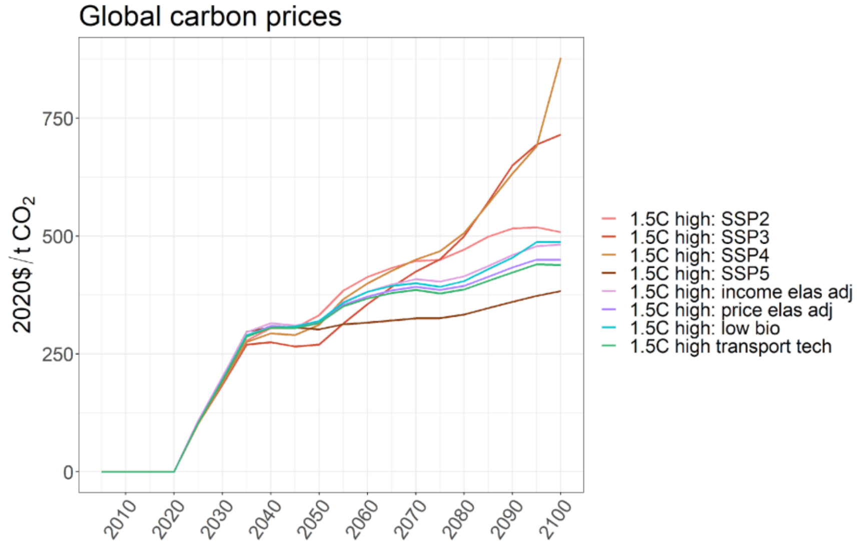

Supplementary Figure 15. Carbon prices across sensitivity scenarios. Global carbon prices, calculated endogenously by GCAM as required to meet the carbon emissions constraint, across sensitivity scenarios. Units are 2020 USD per ton of CO_2. SSP = Shared Socioeconomic Pathway, income elas adj = income elasticity adjustment, price elas adj = price elasticity adjustment, low bio = low bioenergy.

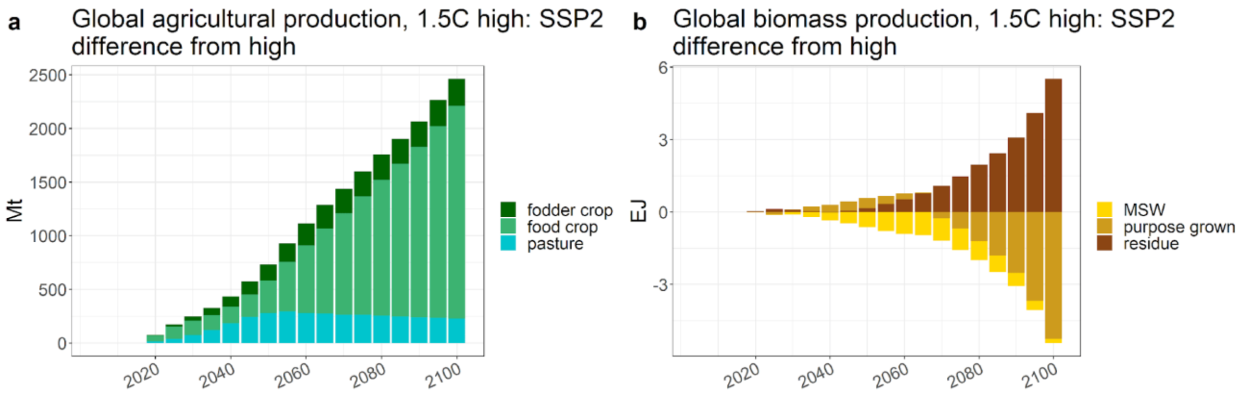

Supplementary Figure 16. Agricultural and biomass production, sensitivity scenarios. (a) Global agricultural production and (b) global biomass production in the Shared Socioeconomic Pathway 2 (SSP2) sensitivity scenario, shown as a difference from the production in the standard high transportation technology scenario. Positive values indicate higher production in the SSP2 sensitivity scenario than in the standard high scenario. MSW = municipal solid waste.

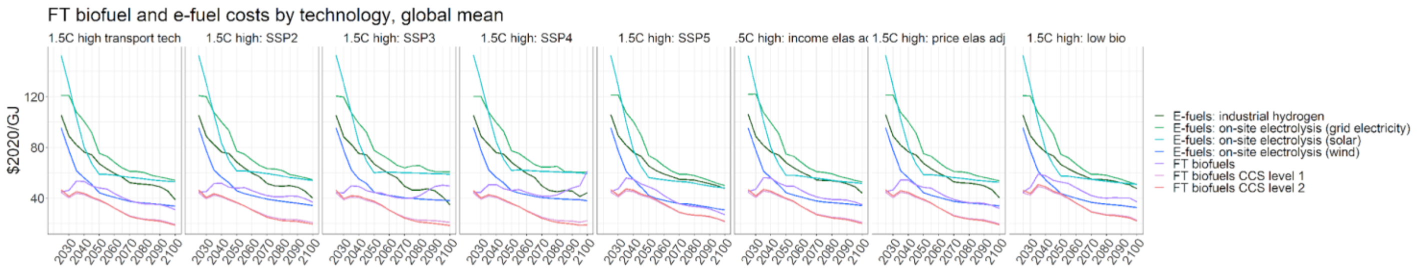

Supplementary Figure 17. FT biofuel and e-fuel costs, sensitivity scenarios. Global mean of Fischer-Tropsch (FT) biofuel and e-fuel costs by production technology in the sensitivity scenarios compared to the standard high transport technology scenario (far left panel). The different carbon capture and storage (CCS) levels indicate varied carbon removal fractions, with level 2 technologies employing carbon capture on a more expansive set of CO_2 sources and thus yielding a higher removal fraction than level 1 technologies. The costs shown in this figure incorporate the effects of carbon prices on the overall cost of the fuel produced. SSP = Shared Socioeconomic Pathway, income elas adj = income elasticity adjustment, price elas adj = price elasticity adjustment, low bio = low bioenergy.

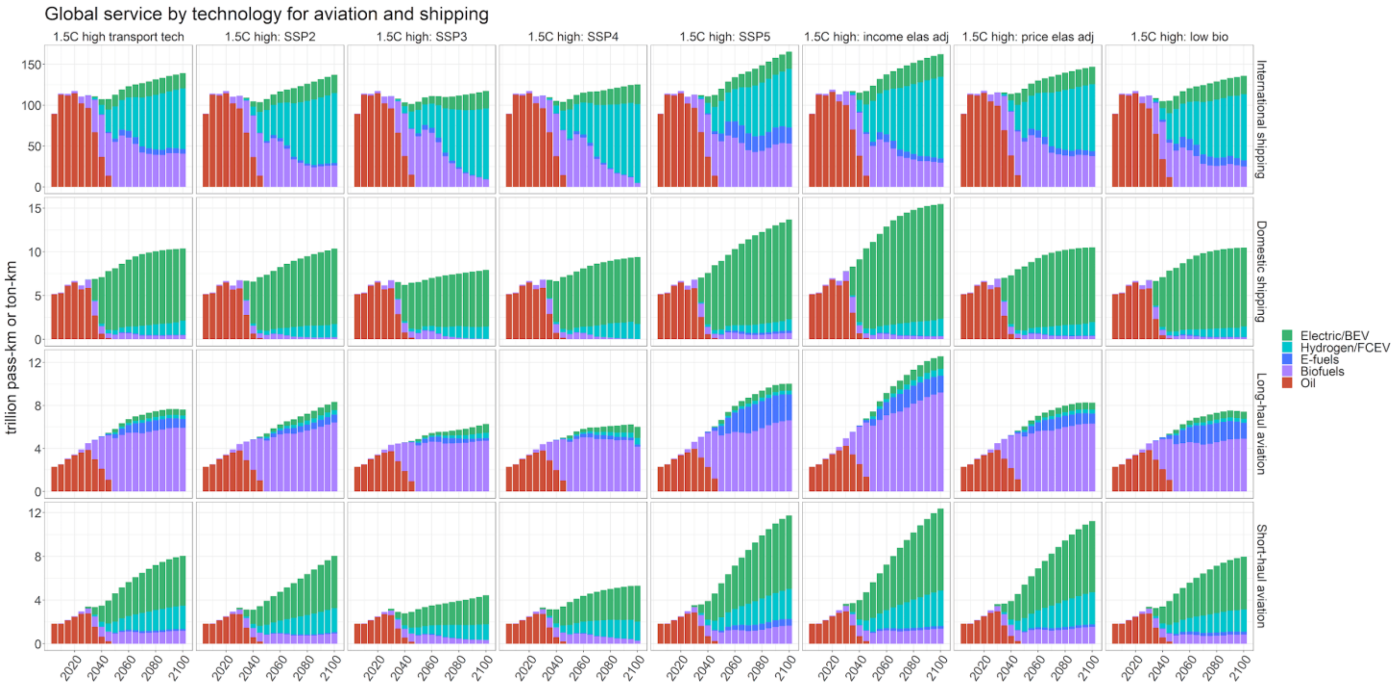

Supplementary Figure 18. Transportation service by technology for aviation and shipping, sensitivity scenarios. Global service provided by technology for international and domestic shipping and long-haul and short-haul aviation in the high transportation technology scenario and the sensitivity scenarios. Note the different y-axis scales and units for each mode; units are trillion passenger-kilometers (pass-km) for aviation and trillion ton-kilometers (ton-km) for shipping. For aviation, hydrogen technologies employ hydrogen combustion turbines, while for shipping, hydrogen fuel cell electric vessels are modeled. SSP = Shared Socioeconomic Pathway, income elas adj = income elasticity adjustment, price elas adj = price elasticity adjustment, low bio = low bioenergy, BEV = battery electric vehicle, FCEV = fuel cell electric vehicle.

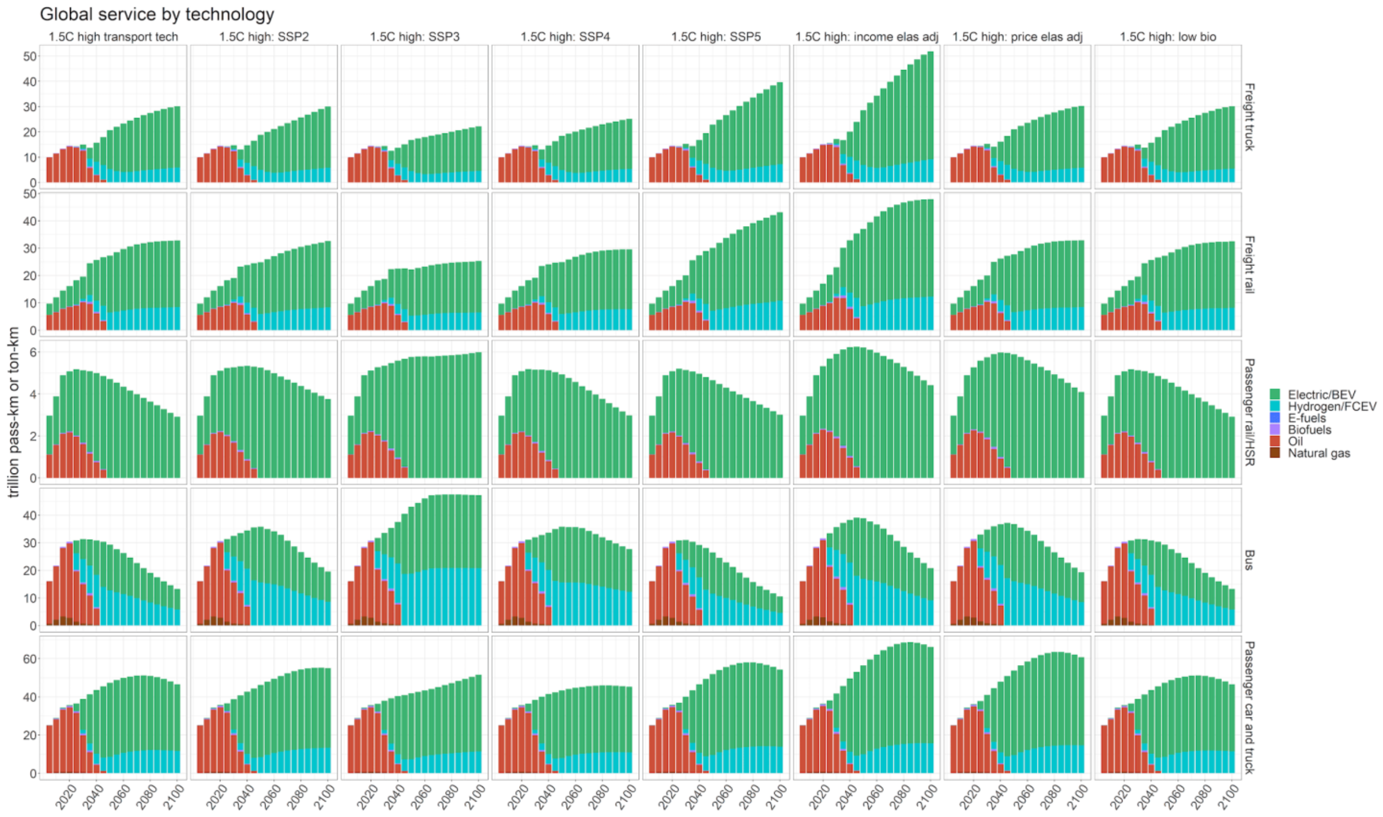

Supplementary Figure 19. Transportation service by technology for modes other than aviation and shipping, sensitivity scenarios. Global service provided by technology for freight trucks, freight rail, passenger rail and high-speed rail (HSR), buses, and passenger cars and trucks in the high transportation technology scenario and the sensitivity scenarios. Note the different y-axis scales and units for each mode; units are trillion passenger-kilometers (pass-km) for passenger modes and trillion ton-kilometers (ton-km) for freight modes. Also note that there is a very small amount of coal-based freight rail service that is not shown in the figure as it phases out by 2025 and provides only a miniscule contribution to global freight rail transport in the preceding periods (0.009 trillion ton-km of service in 2005, 0.007 trillion ton-km in 2010, and 0.002 trillion ton-km in 2015 and 2020). SSP = Shared Socioeconomic Pathway, income elas adj = income elasticity adjustment, price elas adj = price elasticity adjustment, low bio = low bioenergy, BEV = battery electric vehicle, FCEV = fuel cell electric vehicle.

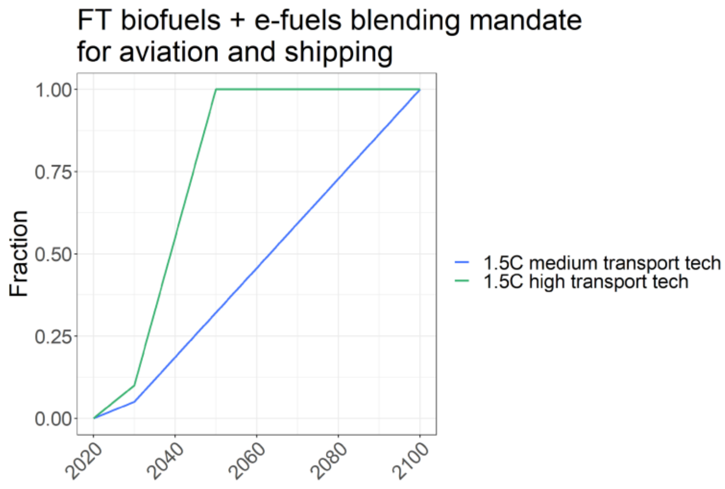

Supplementary Figure 20. Alternative fuel blending mandates for aviation and shipping. Scenario assumptions for the minimum fraction of liquid fuels consumed by aviation and shipping that must be generated from Fischer-Tropsch (FT) biofuels or e-fuels. Only the scenarios with alternative fuel mandates are shown.

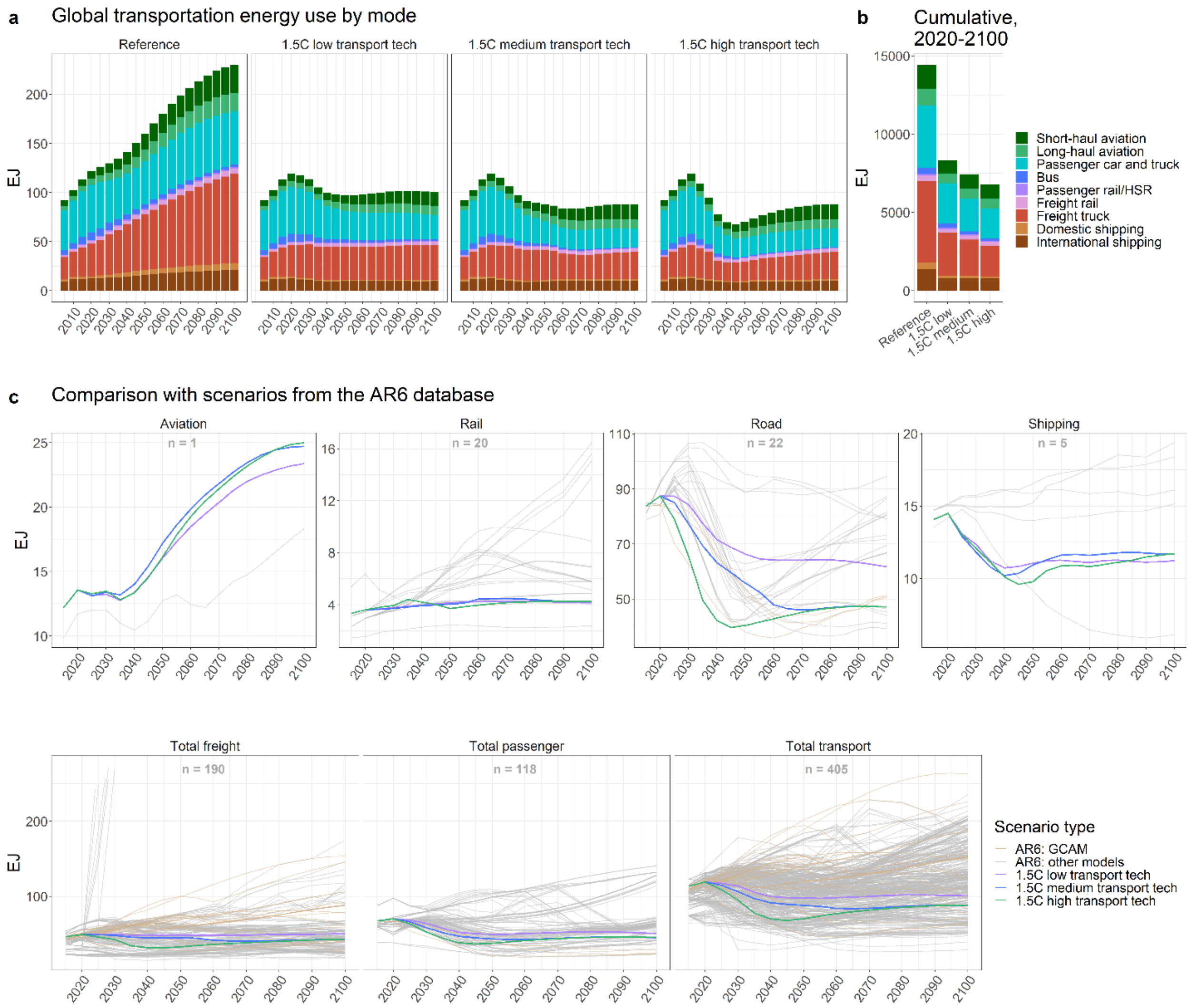

Supplementary Figure 21. Global transportation fuel use by mode. (a) Global transportation sector energy use, by mode, across the four scenarios. (b) Cumulative global transportation sector energy use, by mode, from 2020 to 2100. (c) Global energy use by the full transportation sector and by aggregated groups of modes in our three decarbonization scenarios compared to scenarios in the Sixth Assessment Report (AR6) database that are consistent with limiting end-of-century warming to 1.5°C (with scenarios in the database generated by GCAM shown in brown and scenarios generated by all other models shown in gray). The number of scenarios from the database with results for each subset of transportation modes is indicated by the number in gray at the top of each panel. Note that the GCAM scenarios in the AR6 database employed earlier versions of GCAM than the version used in this study, and thus the scenarios may differ in historical periods due to differences in calibration data and other model updates. Also note the different axis scales for Aviation, Rail, Road, and Shipping in panel (c). HSR = high-speed rail.

Supplementary Tables

Supplementary Table 1. Direct air capture (DAC) cost assumptions per ton of CO_2. Costs are derived from GCAM assumptions for DAC non-energy costs and energy coefficients, as well as endogenously calculated GCAM energy costs, and are linearly interpolated between the years shown in the table.

Technology	Units	2020 cost	2050 cost	2100 cost
High temperature DAC with natural gas	2020$ per tCO_2	447.8	172.3	166.5
Low temperature DAC with heat pumps	2020$ per tCO_2	625.7	291.2	284.3
High temperature DAC with electricity	2020$ per tCO_2	599.6	350.9	337.2

Supplementary Table 2. Cumulative CO_2 emissions, 2020 to 2100, from each transportation mode in each scenario. HSR = high-speed rail.

Mode	Units	Reference	1.5°C low	1.5°C medium	1.5°C high
Bus	Gt CO_2	21.6	14.4	7.6	5.5
Passenger car and truck	Gt CO_2	204.3	110.2	59.7	40.7
Passenger rail/HSR	Gt CO_2	2.9	1.7	1.1	0.6
Short-haul aviation	Gt CO_2	77.0	32.2	19.7	7.9
Long-haul aviation	Gt CO_2	68.4	35.4	20.2	8.0
Freight rail	Gt CO_2	14.3	8.2	6.1	3.2
Freight truck	Gt CO_2	285.7	109.9	71	31.9
Domestic shipping	Gt CO_2	25.5	5.2	3.3	2.0
International shipping	Gt CO_2	87.8	40.9	24.0	13.0
Total	Gt CO_2	787.4	358.1	212.8	112.8

Supplementary Table 3. Cumulative non-CO_2 emissions, 2020 to 2100, from the total transportation sector in each scenario.

Non-CO_2 gas	Units	Reference	1.5°C low	1.5°C medium	1.5°C high
BC	Tg	128.7	26.7	21.1	13.4
CH4	Tg	141.3	62.1	35.1	19.9
CO	Tg	11595.9	2950.4	2209.1	1583.7
N2O	Tg	25.4	12.4	8.6	5.5
NH3	Tg	27	15.6	10.7	7.9
NMVOC	Tg	2272.2	576.1	423.3	309.3
NOx	Tg	3752.2	1050.2	819.7	578.0
OC	Tg	50.6	11.5	8.3	5.4
SO2	Tg	297.8	145.5	104.2	85.1

Supplementary Table 4. Break-even carbon prices for electric and hydrogen-based transportation technologies in selected years. Break-even carbon prices are calculated as the difference in costs (on a per service output basis) between the low carbon technologies and the corresponding standard refined liquids-based technology, divided by their difference in emissions per service output. We only consider direct emissions here; thus, electric and hydrogen-based technologies are assumed to generate zero emissions. Cost values are used from the reference scenario. Results are shown for the US as a representative example, as costs vary regionally, and include values for shipping and aviation as well as for the rail and road modes that are employed in all regions. A 0 value indicates the technology is already cost-competitive without an additional carbon price. BEV = battery electric vehicle, FCEV = fuel cell electric vehicle.

Mode	Technology	Units	2030 break-even carbon price	2050 break-even carbon price
Short-haul aviation	BEV	2020$ per tCO_2	404	296
	Hydrogen	2020$ per tCO_2	2474	337
Long-haul aviation	BEV	2020$ per tCO_2	12309	1357
	Hydrogen	2020$ per tCO_2	14209	1341
Domestic shipping	BEV	2020$ per tCO_2	423	135
	FCEV	2020$ per tCO_2	242	195
International shipping	BEV	2020$ per tCO_2	1320	496
	FCEV	2020$ per tCO_2	411	237
Freight rail	BEV	2020$ per tCO_2	66	58
	FCEV	2020$ per tCO_2	158	123
Heavy truck	BEV	2020$ per tCO_2	876	461
	FCEV	2020$ per tCO_2	580	609
Medium truck	BEV	2020$ per tCO_2	205	156
	FCEV	2020$ per tCO_2	298	356
Light truck	BEV	2020$ per tCO_2	0	0
	FCEV	2020$ per tCO_2	327	252
Passenger rail	Electric	2020$ per tCO_2	0	0
Bus	BEV	2020$ per tCO_2	305	0
	FCEV	2020$ per tCO_2	96	0
Car	BEV	2020$ per tCO_2	8	0
	FCEV	2020$ per tCO_2	659	481
Large car and truck	BEV	2020$ per tCO_2	12	19
	FCEV	2020$ per tCO_2	698	499

Supplementary Table 5. Cumulative service provided by each transportation mode, 2020 to 2100, in each scenario. The "no policy, tech + behavior change" scenario is a comparison scenario that incorporates the economy-wide behavioral and technological changes used in the 1.5°C scenarios (e.g., reduced demand for transportation services, lower population growth), except for the advanced transportation technology assumptions. Thus, the difference between the reference scenario and the "no policy, tech + behavior change" scenario highlights the effect of the non-transportation-technology assumptions on service provision. The differences between the 1.5°C scenarios and the "no policy, tech + behavior change" scenario isolate the effects of the carbon policy and the transportation technology changes. HSR = high-speed rail, pass-km = passenger-kilometers, ton-km = ton-kilometers.

Mode	Units	Reference	No policy, tech + behavior change	1.5°C low	1.5°C medium	1.5°C high
Bus	Trillion pass-km	2700	1962	1990	1996	1996
Passenger car and truck	Trillion pass-km	4710	3833	3719	3757	3763
Passenger rail/HSR	Trillion pass-km	465	339	337	341	345
Short-haul aviation	Trillion pass-km	774	597	422	449	444
Long-haul aviation	Trillion pass-km	856	622	494	520	506
Freight rail	Trillion ton-km	2412	1732	1819	2090	2266
Freight truck	Trillion ton-km	3190	2183	1982	1899	1804
Domestic shipping	Trillion ton-km	1515	1062	487	644	704
International shipping	Trillion ton-km	15646	13659	9471	10001	9889
Total passenger	Trillion pass-km	9506	7354	6960	7063	7055
Total freight	Trillion ton-km	22764	18635	13759	14633	14663

Supplementary Table 6. Cumulative service provided by each transportation mode, 2020 to 2050, in each scenario. HSR = high-speed rail, pass-km = passenger-kilometers, ton-km = ton-kilometers.

Mode	Units	Reference	No policy, tech + behavior change	1.5°C low	1.5°C medium	1.5°C high
Bus	Trillion pass-km	1148	942	956	953	952
Passenger car and truck	Trillion pass-km	1418	1309	1260	1271	1278
Passenger rail/HSR	Trillion pass-km	188	154	152	153	156
Short-haul aviation	Trillion pass-km	172	155	108	112	107
Long-haul aviation	Trillion pass-km	201	174	145	148	147
Freight rail	Trillion ton-km	715	575	598	606	703
Freight truck	Trillion ton-km	745	610	559	561	489
Domestic shipping	Trillion ton-km	396	324	146	178	218
International shipping	Trillion ton-km	4670	4378	3285	3448	3434
Total passenger	Trillion pass-km	3127	2733	2620	2637	2639
Total freight	Trillion ton-km	6526	5887	4588	4792	4845

Supplementary Table 7. Cumulative CO_2 emissions, 2020 to 2100, from each sector in each scenario. LULUCF = land use, land use change, and forestry.

Sector	Units	Reference	1.5°C low	1.5°C medium	1.5°C high
Buildings	Gt CO_2	218.8	57.8	83.3	97.6
CO_2 removal	Gt CO_2	-0.8	-360.5	-370.4	-374.1
Electricity	Gt CO_2	1901.4	34.8	71.7	109.0
Hydrogen	Gt CO_2	70.9	-61.8	-44.0	-40.9
Industry	Gt CO_2	1215.0	203.9	281.0	332.3
LULUCF	Gt CO_2	110.2	-233.0	-233.9	-234.3
Other energy supply	Gt CO_2	393.8	-3.0	-5.0	-7.2
Transportation	Gt CO_2	787.4	358.1	212.8	112.8
Total	Gt CO_2	4696.6	-3.6	-4.5	-4.8

Supplementary Table 8. Cumulative CO_2 emissions, 2020 to 2047 (the year in which net zero CO_2 emissions are achieved in the medium and high scenarios), from each sector in each scenario. The low scenario achieves net zero CO_2 emissions in 2048. LULUCF = land use, land use change, and forestry.

Sector	Units	Reference	1.5°C low	1.5°C medium	1.5°C high
Buildings	Gt CO_2	84.3	38.4	42.3	51.0
CO_2 removal	Gt CO_2	-0.1	-43.4	-43.5	-43.4
Electricity	Gt CO_2	522.0	101.2	110.0	131.2
Hydrogen	Gt CO_2	8.5	-6.7	-4.5	-1.3
Industry	Gt CO_2	384.8	180.8	191.5	214.3
LULUCF	Gt CO_2	103.4	-32.2	-34.7	-37.9
Other energy supply	Gt CO_2	78.9	33.6	31.2	27.0
Transportation	Gt CO_2	237.9	187.3	164.4	112.5
Total	Gt CO_2	1419.7	459.1	456.6	453.4

Supplementary Table 9. Aviation and shipping consumption of FT biofuels and e-fuels. Fraction of all Fischer-Tropsch (FT) biofuels and e-fuels produced that are consumed by aviation and shipping, in selected years.

Scenario	Year	Fraction of FT biofuels and e-fuels consumed by aviation	Fraction of FT biofuels and e-fuels consumed by shipping
1.5°C medium	2025	0.50	0.50
1.5°C medium	2050	0.64	0.36
1.5°C medium	2075	0.69	0.31
1.5°C medium	2100	0.75	0.25
1.5°C high	2025	0.51	0.49
1.5°C high	2050	0.62	0.38
1.5°C high	2075	0.71	0.29
1.5°C high	2100	0.73	0.27

Supplementary Table 10. Fraction of refined liquids supply that comes from biofuels and e-fuels for non-transportation sectors (electricity, industry, buildings, and other energy supply) by year and scenario.

Year	Reference	1.5°C low	1.5°C medium	1.5°C high
2015	0.02	0.02	0.02	0.02
2020	0.02	0.02	0.02	0.02
2025	0.03	0.03	0.03	0.03
2030	0.04	0.06	0.05	0.04
2035	0.05	0.09	0.08	0.07
2040	0.07	0.10	0.09	0.08
2045	0.10	0.14	0.11	0.09
2050	0.13	0.18	0.14	0.11
2055	0.14	0.21	0.15	0.13
2060	0.15	0.22	0.16	0.14
2065	0.14	0.22	0.16	0.14
2070	0.14	0.21	0.15	0.14
2075	0.13	0.21	0.14	0.14
2080	0.12	0.24	0.15	0.14
2085	0.12	0.27	0.15	0.15
2090	0.12	0.30	0.17	0.16
2095	0.11	0.33	0.19	0.18
2100	0.11	0.35	0.21	0.20

Supplementary Methods

Shipping and Aviation Technology Assumptions: Full documentation of the assumptions for advanced technologies, for all transportation modes, is available in the GCAM documentation[1,2]. We include information on the non-fuel costs and energy intensities for shipping and aviation technologies in Supplementary Table 11, as we emphasize transition pathways for those modes in our analysis. Electric and hydrogen-powered aviation and shipping costs are based on existing literature[3–5], where available, or are estimated based on the cost ratio between conventional and advanced technologies for modes with similar drivetrains. Specifically, battery electric aircraft costs, including both capital costs and non-fuel operation and maintenance costs, are obtained from the all-electric aircraft technology outlined in Schäfer et al.[4] This technology is assumed to have a fuel efficiency double that of standard jet engine planes. Hydrogen-powered aircraft are modeled after the technologies outlined in the Destination 2050 report and represent aircraft that combust hydrogen in a turbine[3,5]. This technology is expected to increase capital costs by 31% and non-fuel operation and maintenance costs by 47% relative to conventional aircraft due to the costs associated with the hydrogen storage tank and the fuel distribution system, as well as the larger required aircraft size[3,5]. Hydrogen-powered aircraft are also projected to have longer refueling times than conventional aircraft, decreasing flight cycles by 7%, as well as lowered seating capacity, further decreasing productivity by about 12%[3,5]. For marine shipping vessels, elevated costs for battery electric and fuel cell electric technologies are calculated based on the cost increases associated with the corresponding technologies in freight rail, as ships and rail have similar drivetrain technologies. Specifically, the ratio of battery electric rail costs to conventional rail costs is applied to conventional ship costs to obtain battery electric ship costs (with a comparable calculation performed for fuel cell electric ship costs). For freight rail, advanced technology costs are calculated from the rail technology assessment by the California Air Resources Board[1,6].

Supplementary Table 11. Shipping and aviation non-fuel costs and energy intensities for the United States. Values are interpolated between the years shown in the table. Note that hydrogen aircraft and electric long-haul aircraft are not introduced until 2035 and thus do not have values reported in 2030. Cost values for the United States specifically are reported in this table; costs vary regionally, but the cost differentials between technologies for each mode are comparable across regions. BEV = battery electric vehicle, FCEV = fuel cell electric vehicle.

**Mode**	**Technology**	**Energy intensity (MJ per vkm)**			**Non-fuel cost (2020$ per vkm)**		
		**2030**	**2035**	**2050**	**2030**	**2035**	**2050**
Short-haul aviation	Liquid fuels	280	280	280	16.15	16.63	16.64
	BEV	280	280	280	22.26	22.11	19.61
	Hydrogen	N/A	280	280	N/A	21.89	16.62
Long-haul aviation	Liquid fuels	333	313	313	21.50	22.16	22.16
	BEV	N/A	313	313	N/A	73.68	49.43
	Hydrogen	N/A	313	313	N/A	80.72	44.80
Domestic shipping	Liquid fuels	309	303	297	1.64	1.64	1.64
	Liquid fuels - high efficiency	267	265	261	2.05	1.76	1.67
	BEV	167	166	163	13.64	7.05	6.67
	FCEV	222	221	218	4.36	3.73	2.82
International shipping	Liquid fuels	2072	2062	2031	29.69	29.69	29.69
	Liquid fuels - high efficiency	1658	1649	1625	37.12	31.98	30.27
	BEV	1036	1031	1015	247.46	127.92	121.06
	FCEV	1381	1374	1354	79.19	67.61	51.17

Supplementary Notes

Additional Discussion of Shifting Patterns of Transportation Service Across Scenarios

As explained in the main text, carbon price effects reduce aviation and shipping service output in the decarbonization scenarios, beyond the reductions due to the lowered demand assumptions. However, more aggressive implementation of advanced technologies in the medium and high ambition scenarios allows for some rebounding of aviation and shipping service in those scenarios relative to in the low ambition scenario, as fuel switching is a more feasible option in those scenarios and thus there is a reduced need to rely on demand destruction to meet emissions mitigation goals (Figure 3 in main text and Supplementary Tables 5 and 6). For long-haul and short-haul aviation and international shipping, these differences are relatively small, on the order of 2-7% increases in cumulative service over the century. These modes also provide slightly less service in the high scenario than in the medium scenario, as the more rapid elimination of oil-based fuels in the high scenario makes it more challenging to meet similar levels of service, especially earlier in the century while alternative technologies are still scaling up. For domestic shipping, the high scenario exceeds the medium scenario in service output throughout the century, and differences relative to the low scenario are larger as well. Of the other modes, only freight trucks see comparable reductions in service due to the carbon policy on top of the lowered demand assumptions (Supplementary Figure 7 and Supplementary Tables 5 and 6). Furthermore, unlike all other modes, freight truck service is lower in the medium and high scenarios than in the low scenario. These declines are more than offset by increases in freight rail service, however, suggesting that faster electrification of freight rail paired with early phase-outs of fossil fuels in freight road transport leads to mode shifting to the relatively cheaper to decarbonize rail service.

Additional Discussion of Sensitivity Scenarios

Considering the transportation modes separately, the relative shares of each of the advanced technologies are largely consistent across the standard high ambition scenario and the sensitivity scenarios (Supplementary Figures 18 and 19). Biofuel use shows one of the largest variations between the scenarios; as discussed in the main text, the differences between the SSP2 scenario and the standard high ambition scenario likely arise from increased land competition between biomass production and food crop production in the SSP2 scenario that leads to reduced biofuel use in transportation. In the SSP3 and SSP4 scenarios, biofuel use in international shipping in particular declines notably relative to the standard high ambition scenario, largely replaced by hydrogen consumption, as the elevated carbon prices in those scenarios make hydrogen-based shipping more cost-favorable. In contrast, in the SSP5 scenario, the shares of both biofuel- and e-fuel-based service increase for international shipping and long-haul aviation, as hydrogen and electricity are more widely used by other transport modes and other economic sectors and e-fuels achieve their lowest costs. An elevated constraint on economy-wide bioenergy use also most notably impacts international shipping and long-haul aviation. In this scenario, hydrogen, electricity, and e-fuels all contribute to replacing biofuels in international shipping, while for long-haul aviation, the deficit in biofuels is largely compensated for by e-fuels; by 2100, e-fuels constitute 23% of the total alternative fuel mix for long-haul aviation and provide 20% of total service for the mode.

In the income and price elasticity adjustment scenarios, overall biofuel consumption by transport is comparable to or greater than that in the standard high ambition scenario, but the allocation of biofuels between modes differs. As long-haul aviation depends heavily on biofuels in its decarbonization

pathway, it must meet most of its increases in demand in the elasticity adjustment scenarios using biofuels. Elevated biofuel use in aviation reduces its availability for other transportation modes, most notably international shipping, where hydrogen and electricity must then both make up the deficit in biofuel use and provide for the additional service due to the increased demand. These dynamics are most visible in the income elasticity adjustment scenario, but also occur to a lesser extent in the price elasticity adjustment scenario.

Supplementary References

1. Kyle, P., Fuhrman, J., Wolfram, P., O'Rourke, P. & Kholod, N. Core Model Proposal #359: Hydrogen and transportation technology update. (2022).

2. Mishra, G. S. *et al. Transportation Module of Global Change Assessment Model (GCAM)*. (2013).

3. Royal Netherlands Aerospace Centre. *Destination 2050: A Route To Net Zero European Aviation*. https://reports.nlr.nl/server/api/core/bitstreams/c9002b7e-224f-420c-b6da-ab6aecd48ea2/content (2021).

4. Schäfer, A. W. *et al.* Technological, economic and environmental prospects of all-electric aircraft. *Nat Energy* **4**, 160–166 (2019).

5. McKinsey & Company. *Hydrogen-powered aviation: A fact-based study of hydrogen technology, economics, and climate impact by 2050*. https://www.euractiv.com/wp-content/uploads/sites/2/2020/06/20200507_Hydrogen-Powered-Aviation-report_FINAL-web-ID-8706035.pdf (2020).

6. California Air Resources Board. *Technology Assessment: Freight Locomotives*. https://ww2.arb.ca.gov/sites/default/files/classic/msprog/tech/techreport/final_rail_tech_assessment_11282016.pdf (2016).
