# Supplementary material for: Integrated assessment modeling of a zero-emissions global transportation sector
Source: Nat Commun. 2024 May 24;15:4439. doi: 10.1038/s41467-024-48424-9 (PMC11126718; doi:10.1038/s41467-024-48424-9)
Supplement: Supplementary file 3 — Reporting Summary [file 41467_2024_48424_MOESM3_ESM.pdf]

nature portfolio

Corresponding author(s): Haewon McJeon

Last updated by author(s): Feb 19, 2024

Reporting Summary

Nature Portfolio wishes to improve the reproducibility of the work that we publish. This form provides structure for consistency and transparency in reporting. For further information on Nature Portfolio policies, see our Editorial Policies and the Editorial Policy Checklist.

Statistics

For all statistical analyses, confirm that the following items are present in the figure legend, table legend, main text, or Methods section.

n/a	Confirmed	
☒	☐	The exact sample size (*n*) for each experimental group/condition, given as a discrete number and unit of measurement
☒	☐	A statement on whether measurements were taken from distinct samples or whether the same sample was measured repeatedly
☒	☐	The statistical test(s) used AND whether they are one- or two-sided<br>*Only common tests should be described solely by name; describe more complex techniques in the Methods section.*
☒	☐	A description of all covariates tested
☒	☐	A description of any assumptions or corrections, such as tests of normality and adjustment for multiple comparisons
☒	☐	A full description of the statistical parameters including central tendency (e.g. means) or other basic estimates (e.g. regression coefficient) AND variation (e.g. standard deviation) or associated estimates of uncertainty (e.g. confidence intervals)
☒	☐	For null hypothesis testing, the test statistic (e.g. *F*, *t*, *r*) with confidence intervals, effect sizes, degrees of freedom and *P* value noted<br>*Give P values as exact values whenever suitable.*
☒	☐	For Bayesian analysis, information on the choice of priors and Markov chain Monte Carlo settings
☒	☐	For hierarchical and complex designs, identification of the appropriate level for tests and full reporting of outcomes
☒	☐	Estimates of effect sizes (e.g. Cohen's *d*, Pearson's *r*), indicating how they were calculated

Our web collection on statistics for biologists contains articles on many of the points above.

Software and code

Policy information about availability of computer code

Data collection	All data was collected from computer model simulations of the Global Change Analysis Model
Data analysis	All analysis was conducted with R 3.3

For manuscripts utilizing custom algorithms or software that are central to the research but not yet described in published literature, software must be made available to editors and reviewers. We strongly encourage code deposition in a community repository (e.g. GitHub). See the Nature Portfolio guidelines for submitting code & software for further information.

Data

Policy information about availability of data

All manuscripts must include a data availability statement. This statement should provide the following information, where applicable:

- Accession codes, unique identifiers, or web links for publicly available datasets
- A description of any restrictions on data availability
- For clinical datasets or third party data, please ensure that the statement adheres to our policy

Scripts used to process and analyze GCAM output data and produce figures are available at https://github.com/sspeizer/gcam-cwf-trn-analysis.

Research involving human participants, their data, or biological material

Policy information about studies with human participants or human data. See also policy information about sex, gender (identity/presentation), and sexual orientation and race, ethnicity and racism.

Reporting on sex and gender	Not applicable
Reporting on race, ethnicity, or other socially relevant groupings	Not applicable
Population characteristics	Not applicable
Recruitment	Not applicable
Ethics oversight	Not applicable

Note that full information on the approval of the study protocol must also be provided in the manuscript.

Field-specific reporting

Please select the one below that is the best fit for your research. If you are not sure, read the appropriate sections before making your selection.

☐ Life sciences ☐ Behavioural & social sciences ☒ Ecological, evolutionary & environmental sciences

For a reference copy of the document with all sections, see nature.com/documents/nr-reporting-summary-flat.pdf

Ecological, evolutionary & environmental sciences study design

All studies must disclose on these points even when the disclosure is negative.

Study description	This analysis combines publicly available technology data with Global Change Analysis Model to explore how CO2 emissions change in the future.
Research sample	The research sample of this study is the direct model output of global emission profiles and energy use in GCAM for 2005-2100. This data is obtained for each of the scenarios described in the main text.
Sampling strategy	The study sample contains all 32 GCAM regions and all modeling periods (5-year intervals from 2005 to 2100) for all scenarios. No sample was excluded. All data was collected from the GCAM simulations.
Data collection	Data was collected via GCAM model output by the authors.
Timing and spatial scale	Data for the study periods is modeled every 5 years between 2005 and 2100 at 32 region scale. No additional data collection was performed outside GCAM simulations.
Data exclusions	No data was excluded from this study.
Reproducibility	All model input data files will be provided in a Github repository upon publication. GCAM is a deterministic model, such that all results are reproducible. Model output data supporting this analysis will also be deposited in the data repository upon publication.
Randomization	Randomization is not relevant for this study as each scenario was defined deterministically and the initialization conditions would not change simulation results.
Blinding	Blinding is not relevant to this study, as all scenarios in this study are simulated through a deterministic simulation model with no potential placebo effect.

Did the study involve field work? ☐ Yes ☒ No

Reporting for specific materials, systems and methods

We require information from authors about some types of materials, experimental systems and methods used in many studies. Here, indicate whether each material, system or method listed is relevant to your study. If you are not sure if a list item applies to your research, read the appropriate section before selecting a response.

Materials & experimental systems

n/a	Involved in the study
☒	☐ Antibodies
☒	☐ Eukaryotic cell lines
☒	☐ Palaeontology and archaeology
☒	☐ Animals and other organisms
☒	☐ Clinical data
☒	☐ Dual use research of concern
☒	☐ Plants

Methods

n/a	Involved in the study
☒	☐ ChIP-seq
☒	☐ Flow cytometry
☒	☐ MRI-based neuroimaging

Plants

Seed stocks	Not applicable
Novel plant genotypes	Not applicable
Authentication	Not applicable
